# The pseudouridine synthase PUSL1 modifies U39 of mitochondrial tRNAs and is linked to human neurological phenotypes

**DOI:** 10.1093/nar/gkag805

**Published:** 2026-08-14

**Authors:** Pedro Rebelo-Guiomar, Nadav Kra-Oz, Christopher Powell, Guillaume Jouret, Morgane Plutino, Veronique Paquis-Flucklinger, Schraga Schwartz, Aldema Sas-Chen, Michal Minczuk

**Affiliations:** MRC Mitochondrial Biology Unit, University of Cambridge, Cambridge CB2 0XY, United Kingdom; The Shmunis School of Biomedicine and Cancer Research, The George S. Wise Faculty of Life Sciences, Tel Aviv University, Tel Aviv, 69978, Israel; MRC Mitochondrial Biology Unit, University of Cambridge, Cambridge CB2 0XY, United Kingdom; National Center of Genetics (NCG), Laboratoire National de Santé (LNS), Dudelange 3555, Luxembourg; Department of Medical Genetics, Reference Centre for Mitochondrial Diseases, Nice Teaching Hospital, 06200 Nice, France; Department of Medical Genetics, Reference Centre for Mitochondrial Diseases, Nice Teaching Hospital, 06200 Nice, France; IRCAN, UMR 7284/INSERM U1081/UCA, School of Medicine, Nice Côte d’Azur University, 06107 Nice, France; Department of Molecular Genetics, Weizmann Institute of Science, Rehovot, 7610001, Israel; The Shmunis School of Biomedicine and Cancer Research, The George S. Wise Faculty of Life Sciences, Tel Aviv University, Tel Aviv, 69978, Israel; MRC Mitochondrial Biology Unit, University of Cambridge, Cambridge CB2 0XY, United Kingdom; Department of Clinical Neurosciences, University of Cambridge, Cambridge CB2 0QQ, United Kingdom

## Abstract

Variants in the mitochondrial and nuclear genomes are linked to a wide range of human disorders marked by impaired mitochondrial function. Among these disorders, there is a growing number of patients with variants affecting mitochondrial RNA biology. Mitochondrial transcripts are pseudouridylated, and some enzymes responsible for this modification—pseudouridine synthases (PUS)—have been identified. Although known as the ‘fifth nucleotide’ owing to its high abundance in transcripts, the exact cellular role of pseudouridine is still unclear. Here, we expand the group of mitochondrial PUS enzymes by demonstrating that the protein encoded by *PUSL1* is an active pseudouridine synthase with mitochondrial localization. Nucleotide-resolution pseudouridine mapping (mito-Ψ-Seq) followed by primer extension analysis showed that PUSL1 selectively modifies universal position 39 of all mitochondrial transfer RNAs (tRNAs) with a uridine residue in this position. Two newly described clinical *PUSL1* variants, c.704G > A (p.Arg235Gln) and c.634del, p.Glu212Argfs*26, were functionally studied, presenting defects in pseudouridylation of mt-tRNA position 39 in patient-derived material, corroborating the association of this enzyme with human pathology. Our data show that *PUSL1* regulates mitochondrial RNA post-transcriptional processing and its dysfunction and could be associated with neurological phenotypes.

## Graphical Abstract

**Graphical Abstract:**
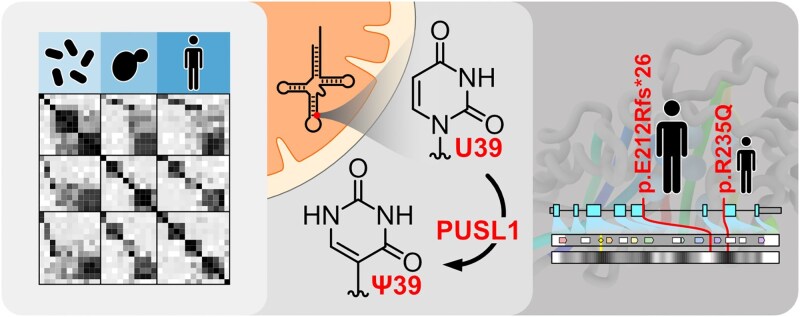

## Introduction

The relevance of the epitranscriptome in regulating cellular functions has been expanding. Development and improvement of high-throughput and nucleotide-resolution technologies is creating opportunities to better understand this conserved yet versatile mode of regulation through transcript modification. Present across different transcript classes and kingdoms of life, pseudouridine (Ψ) is the most abundant post-transcriptionally modified ribonucleotide, owning it the title of ‘fifth nucleotide’ [1]. This uridine (U) isomer is produced by cleavage of the C1’–N1 glycosidic bond, which is replaced by an unusual planar C–C glycosidic bond formed between C1’ of the ribose and C5 of uracil. As a direct consequence, local stabilization is enhanced both by base stacking and by N1 interacting with the 5′ phosphate group of the backbone via hydrogen bonds with a water molecule, which restrains the conformation of the base [2]. Regardless of these changes, the Watson–Crick edge remains unchanged and thus uridine and pseudouridine share the same base pairing propensities.

RNA pseudouridylation is performed by standalone enzymes, as well as small nucleolar RNA (snoRNA)-guided RNPs (box H/ACA). Pseudouridine synthases (PUS) can be grouped in five families (RluA, RsuA, TruA, TruB, TruD) based on the bacterial system that share limited sequence similarity although presenting a common β-sheet core structure. This fold is bisected by the catalytic cleft containing a highly conserved aspartate residue, and variably appended with elements that may contribute to modulating substrate specificity and protein–protein interactions [3–12]. One half of the β-sheet is conserved throughout evolution in its arrangement of strands, forming an α/β-sandwich RNA recognition motif (RRM) [13], while the other half presents higher diversity in the organization of its strands between different families, but not within the same family.

The human genome encodes thirteen putative PUS in this class, some with cytosolic or/and mitochondrial localization. *PUS1* encodes two protein isoforms, with cytosolic and mitochondrial localization, which modify residues U27 and U28 of transfer RNAs (tRNAs) in their respective cellular compartment [4, 13–18]. Furthermore, this pseudouridine synthase is clinically relevant as it is implicated in the pathogenic mechanism of the mitochondrial disorder MLASA (myopathy, lactic acidosis, sideroblastic anaemia) [19–25].

Three more human PUS—TRUB2, RPUSD3, RPUSD4—have been demonstrated to be present in mitochondria and to act on mitochondrial RNAs (mtRNAs) [26–28]. It has been hypothesized that these proteins, alongside FASTKD2, NGRN and WBSCR16, form a functional module involved in mitoribosome biogenesis, required for oxidative phosphorylation (OxPhos) and thus cellular respiration.

Presence of pseudouridine across virtually all kingdoms of life, different transcript classes, and its involvement in human pathogenesis reflect the relevance and essentiality of this not yet fully understood RNA modification. Here, we characterize a novel pseudouridine synthase, PUSL1, demonstrating its cellular localization, enzymatic activity and targets, showing that this enzyme is active in the mitochondrial matrix, where it modifies all mt-tRNAs containing a uridine residue in universal position 39. Although no appreciable effects on mitochondrial translation in homeostatic conditions are observed in cultured cells, the modification introduced by PUSL1 may assume a different relevance in the context of organisms and potentially also under non-homeostatic conditions. Interaction with other genes is also plausible, possibly playing a modulatory role in polygenic disorders.

## Materials and methods

### Bioinformatics

Reference protein sequences of *Escherichia coli, Saccharomyces cerevisiae*, and *Homo sapiens* PUS were obtained from UniProt [29] and aligned using Clustal Omega [30] or BLAST [31], from which the similarity score matrixes were obtained. Pathogenicity scores were obtained from CADD [32], PolyPhen-2 [33], SIFT [34], and ClinVar [35]. Evolutionary constraints were obtained from Aminode [36]. Allele frequencies were obtained from gnomAD [37]. Protein structural models were obtained from PDB [38], and predictions from AlphaFold2 [39] and Alphafold3 [40]. Structures were curated and aligned with ChimeraX [41]. 3D-based protein search was performed using FoldSeek [42], with query models predicted by AlphaFold2, search in the AlphaFold/Proteome v4 database, and informed by TM-score.

### Cell culture

HeLa and HEK 293T Flp-In T-Rex (Thermo Fisher Scientific) were routinely maintained in high glucose Dulbecco’s modified Eagle’s medium (DMEM) with GlutaMAX and sodium pyruvate (Gibco) supplemented with 10% Fetal Bovine Serum (FBS; Gibco), 1× penicillin–streptomycin (Gibco), unless otherwise mentioned. All cells were kept at 37°C and in a 5% CO_2_ atmosphere.

### Generation of cell lines

HEK 293T Flp-In T-Rex cells were transiently transfected with two pSpCas9(BB)-2A-GFP (Addgene, #48138) plasmids encoding sgRNAs to *PUS1* (CCAACAGCGCGCGAAGCTGG, TGTTGGGAGCCTTCGGACGG) or *PUSL1* (GGCCGGCGCCGAACTCATGG, GCGCGCGCGCACGGAGCCTG), by electroporation (Cell Line Nucleofector Kit V, Lonza) and according to the manufacturer’s instructions. Cells expressing GFP were sorted into individual wells and allowed to recover in a 1:1 mix of conditioned and fresh medium. Clones were screened for the presence of the protein encoded in the targeted gene by immunoblotting. Candidate lines were further screened by TOPO sequencing of products of PCR amplification over the targeted genomic regions.

Knock-out cells were complemented with complementary DNAs (cDNAs) containing a C-terminal FLAG tag, encoded in pcDNA5/FRT/TO (Invitrogen) and co-transfected with pOG44 (Stratagene) using Lipofectamine 2000 reagent (Invitrogen). Cells where the cDNA was successfully integrated were selected using 100 μg ml^−1^ hygromycin, and resistant clones were tested for doxycycline-inducible (50 ng ml^−1^, 72 h) expression of the FLAG-tagged protein of interest by immunoblotting.

### Cell proliferation assay

Cells were plated in glucose-free DMEM (Gibco) supplemented with 10% dialysed FBS (Sigma–Aldrich), 1× GlutaMAX (Gibco), 1 mM sodium pyruvate (Gibco), 1× penicillin–streptomycin (Gibco), and either 4.5 g l^−1^ glucose (high glucose), 0.45 g l^−1^ glucose (low glucose), or 0.9 g l^−1^ galactose. Chloramphenicol was omitted or added to a final concentration of 50, 100, 150, and 200 μg ml^−1^. Cell confluence was measured every 4 hours using the Incucyte S3 Live-Cell Analysis System (Essen Bioscience) and analysed with the associated software. Maximum proliferation rate was determined for each imaging field by applying the first derivative of measured confluence over time and determining the maximum value in the region of exponential growth. Samples incubated in galactose medium containing chloramphenicol presented non-proliferating cells; to avoid artefactual interpretations and reduce inherent noise, the average of the first derivative was considered instead. Statistical significance was determined using the one-way ANOVA test with Tuckey adjustment for multiple comparisons.

### Immunocytochemistry

cDNAs were cloned into pmaxGFP (Amaxa) and transfected into HeLa cells using Lipofectamine 2000 (Invitrogen). Cells were grown on collagen-coated coverslips (Corning), washed and fixed for 15 min in 4% formaldehyde. Next, coverslips were incubated in 1% Triton X-100 for 10 min, blocked with 5% FBS (Gibco) for 1 h, and incubated with anti-TOM20 (sc-11415, Santa Cruz) for 1h. After being washed, coverslips were incubated with AF594-conjugated anti-rabbit IgG (A11012, Molecular Probes) for 1 h, after which they were washed and mounted on glass slides with ProLong Antifade Mountant with 4′,6-diamidino-2-phenylindole (DAPI) (Molecular Probes). Images were acquired with a LSM 880 confocal microscope (Zeiss), and processed using Fiji [43].

### Subcellular fractionation

Cultured cells were disrupted in homogenization buffer [10 mM Tris–HCl, pH 7.4, 600 mM mannitol, 1 mM ethylenediaminetetraacetic acid (EDTA), 1 μM PMSF, 1× cOmplete protease inhibitor cocktail (Roche), 0.1% bovine serum albumin (BSA)] using a glass-glass Dounce homogenizer. Debris were removed by centrifugation at 400 × *g* for 10 min. Mitochondria were recovered from the supernatant by centrifugation at 10 000 × *g* for 10 min. Mitochondria were resuspended in homogenization buffer without BSA and incubated for 30 min either with proteinase K (New England Biolabs) at a ratio of 4 μg per 1 mg of total mitochondrial protein, 2% Triton X-100, or proteinase K and Triton X-100. Mitochondria treated only with proteinase K were washed twice with homogenization buffer without BSA and recovered by centrifugation at 10 000 × *g* for 10 min. All steps were carried out at 4°C.

### Immunoblotting

Samples were lysed in 50 mM Tris–HCl, pH 7.4, 150 mM NaCl, 1 mM EDTA, 1% Triton X-100, 1× cOmplete protease inhibitor cocktail (Roche) for 20 min at 4°C, clarified by centrifugation at 5000 × *g* for 5 min at 4°C, and then mixed with LDS sample buffer (Novex) supplemented with DTT before being incubated at 95°C for 5 min and loaded in polyacrylamide gels. After dry transfer of proteins to nitrocellulose membranes, these were blocked with 5% skimmed milk in phosphate-buffered saline (PBS) supplemented with 0.1% Triton X-100 and probed with primary antibodies overnight, at 4°C. After washing, membranes were probed with the respective HRP-conjugated secondary antibodies for 1 h, washed again and developed using ECL (GE Healthcare). Images were processed using Fiji [43].

### RNA extraction for mito-Ψ-Seq library preparation

RNA was extracted from parental HEK 293 T Flp-In T-REx, *PUSL1* knock-out, and complemented *PUSL1* knock-out cells. For each condition, 4 biological replicates were obtained (four 10-cm plates for each genetic condition; total of 12 × 10 cm plates for the entire experiment) after exposure to 50 ng/ml doxycycline for 2 days. Mitochondria isolation and RNA extraction were conducted as previously described [44]. In brief, cells were resuspended in mannitol-containing mitochondria extraction buffer and incubated on ice for 20 min. Cells were then homogenized by running the suspension 15 times through a 25 G needle, followed by a series of low- and high-speed centrifugations. The resulting pellet, representing the mitochondria-enriched fraction, was used as input for RNA purification using TRI Reagent (Thermo Fischer Scientific) according to the manufacturer’s instructions.

### Mito-Ψ-Seq library preparation and sequencing

Strand-specific libraries were generated on the basis of previously described protocols [44, 45]. In brief, for each sample, 200 ng of mitochondria-enriched RNA was fragmented and then subjected to FastAP Thermosensitive Alkaline Phosphatase (Thermo Fischer Scientific) and Turbo DNase (Thermo Fischer Scientific) treatments, followed by a 3′ ligation of RNA adaptor 1 using T4 ligase. Ligated RNA was treated with N-cyclohexyl-N’-(β-[N-methylmorpholino]ethyl)carbodiimide p-toluenesulfonate salt (CMC) followed by reverse transcription using SuperScript III Reverse Transcriptase (Thermo Fischer Scientific). The resulting cDNA was subjected to a 3′ligation with adaptor 2 using T4 ligase. The single-stranded cDNA product was then PCR amplified for 9–12 cycles. Libraries were sequenced on a NextSeq 500 platform (Illumina) generating short paired-end reads.

### Identification of putative pseudouridine sites

A reference genome for alignment of reads was generated on the basis of chrM from the GENCODE release 32 (GRCh38.p13). Reads were mapped using the STAR aligner [46] with—alignIntronMax set to 1. To identify pseudouridylated sites, coverage and number of reads starting and ending at each position were counted using bam2ReadEnds.R [47]. The RT stops generated by CMC one nucleotide downstream of the modified site were utilized to quantify pseudouridine level. Hence, for each position, an RT-stop ratio was calculated by dividing the number of reads stopping at the downstream position by the coverage at that position. The significance of changes in pseudouridine level between experimental conditions was assessed using a generalized linear mixed-effects model (GLMM), by using the ‘glmer’ function from the ‘lme4’ package in R [48, 49]. The processed data depicting RT-stop scores is found in this paper’s Supplemental Information (Supplementary Data S1).

### CMC/HO^–^ RT-PEx

RNA was isolated from cultured cells using the TRIzol reagent (Thermo Fisher Scientific) according to the manufacturer’s instructions and processed according to a variation of the protocol by Bakin and Ofengand [50]: 1 μg of total RNA was used, nucleic acids were precipitated overnight at −80°C, recovered by centrifugation at 12 000 × *g* for 30 min at 4°C and allowed to air dry, and alkaline treatment proceeded for 2 h in the described conditions. Oligonucleotides (mt-tRNA^Gly^: 5′-TTGAATGTTGTCAAAACTAG-3′, mt-tRNA^Phe^: 5′-TGGGGTGATGTGAGCCCGTC-3′, mt-tRNA^Cys^: 5′-CAGGTTTGAAGCTGCTTCTT-3′, mt-tRNA^Met^: 5′-TAGTACGGGAAGGGTAT-3′) were end-labelled with [γ-^32^P]-ATP (Hartmann Analytic) and hybridized to total RNA in annealing buffer (10 mM Tris–HCl, pH 8.0, 1 mM EDTA) by incubation at 80°C for 2 min, a −0.02°C s^−1^ ramp to 45°C, 45°C for 1 min, and a final −0.02°C s^−1^ ramp to 4°C. First-strand cDNA synthesis was performed using the Omniscipt RT kit (Quiagen) and incubating the reverse transcription reactions at 37°C for 1 h, after which, Gel Loading Buffer II (Thermo Fisher Scientific) was added and samples loaded on a cast 10% polyacrylamide sequencing gel [7.7 M urea, 10% acrylamide/bis-acrylamide (19:1), 89 mM Tris Base, 28.5 mM taurine, 0.5 mM EDTA; ammonium persulfate and TEMED were used to initiate polymerization]. The polymerized gel was pre-warmed to 50°C in 89 mM Tris Base, 28.5 mM taurine, 0.5 mM EDTA, samples were loaded and separated at 50 W for 1 h. The gel was transferred to blotting paper and dried overnight at 80°C. Autoradiography was performed with a phosphor screen (GE Healthcare) and images were processed using Fiji [43].

### Labelling of *de novo* synthesized proteins

Cells were incubated in methionine/cysteine-free DMEM (Gibco) for 10 min, and then in methionine/cysteine-free DMEM supplemented with 5% dialysed FBS (Sigma–Aldrich), 1× GlutaMAX (Gibco), 1 mM sodium pyruvate, 96 μg ml^−1^ L-cystine for 20 min, after which 100 μg ml^−1^ emetine were added and incubated for 30 min. Labelling with 100 μCi ml^−1^ of [^35^S]-L-methionine (Perkin Elmer) took place at 37°C for 30 min. Cells were washed in PBS and lysed by addition of 0.1% DDM, 1.7 U μl^−1^ benzonase (Merk Millipore) and 1× cOmplete protease inhibitor cocktail (Roche). Lysate components were separated in 10%–20% Tris-glycine SDS–PAGE gels, which were stained with Coomassie Brilliant Blue before being dried. Autoradiography was performed by exposing the gel to a phosphor screen and developed using a Typhoon Biomolecular Imager (GE Healthcare). Densitometry profiles and baseline-corrected quantification were performed using Fiji [43].

### Whole Exome Sequencing

Genomic DNA was captured using Agilent in-solution enrichment methodology with a biotinylated oligonucleotide probe library (SureSelect XT Clinical Reasearch Exome, 54 Mb, Agilent), followed by paired-end 75 bases massively parallel sequencing on Illumina HiSeq4000 [51]. Sequence capture, enrichment and elution were performed according to manufacturer’s instruction and protocols (SureSelect, Agilent) without modification except for library preparation performed with NEBNext Ultra kit (New England Biolabs).

Base calling was performed using the Real-Time Analysis software sequence pipeline (2.7.6). Sequence reads were mapped to the human genome build (hg19/GRCh37) using Elandv2e (Illumina, CASAVA1.8.2). CASAVA1.8.2 was used to call single-nucleotide variants (SNVs) and short insertions/deletions (max. size: 300 nt), taking into account all reads per position. SNVs and indels with Q(SNPs) < 10 and Q(Indel) < 20, or regions with low mappability (QVCutoff < 90) were filtered out. These steps were performed by IntegraGen SA (Evry, France). Annotation and prioritization (filtering) of the variants were performed by Cartagenia Bench NGS Lab (Agilent, Santa Clara, CA, USA). The variant in the PUSL1 gene was identified by applying the following filtering scheme: from a total of 44 746 variants identified in the patient exome, (i) exclusion of polymorphic variants with a minor allele frequency ≥1 in variant databases, (ii) selection of nonsynonymous, canonical splice-site changes and indel variants, (iii) selection of homozygous variants due to parental consanguinity. Ultimately, eight variants remained in the filter. None concerned a gene previously identified in a rare disease.

## Results

### Phylogenetic and structural analysis of pseudouridine synthases

**Figure 1.**
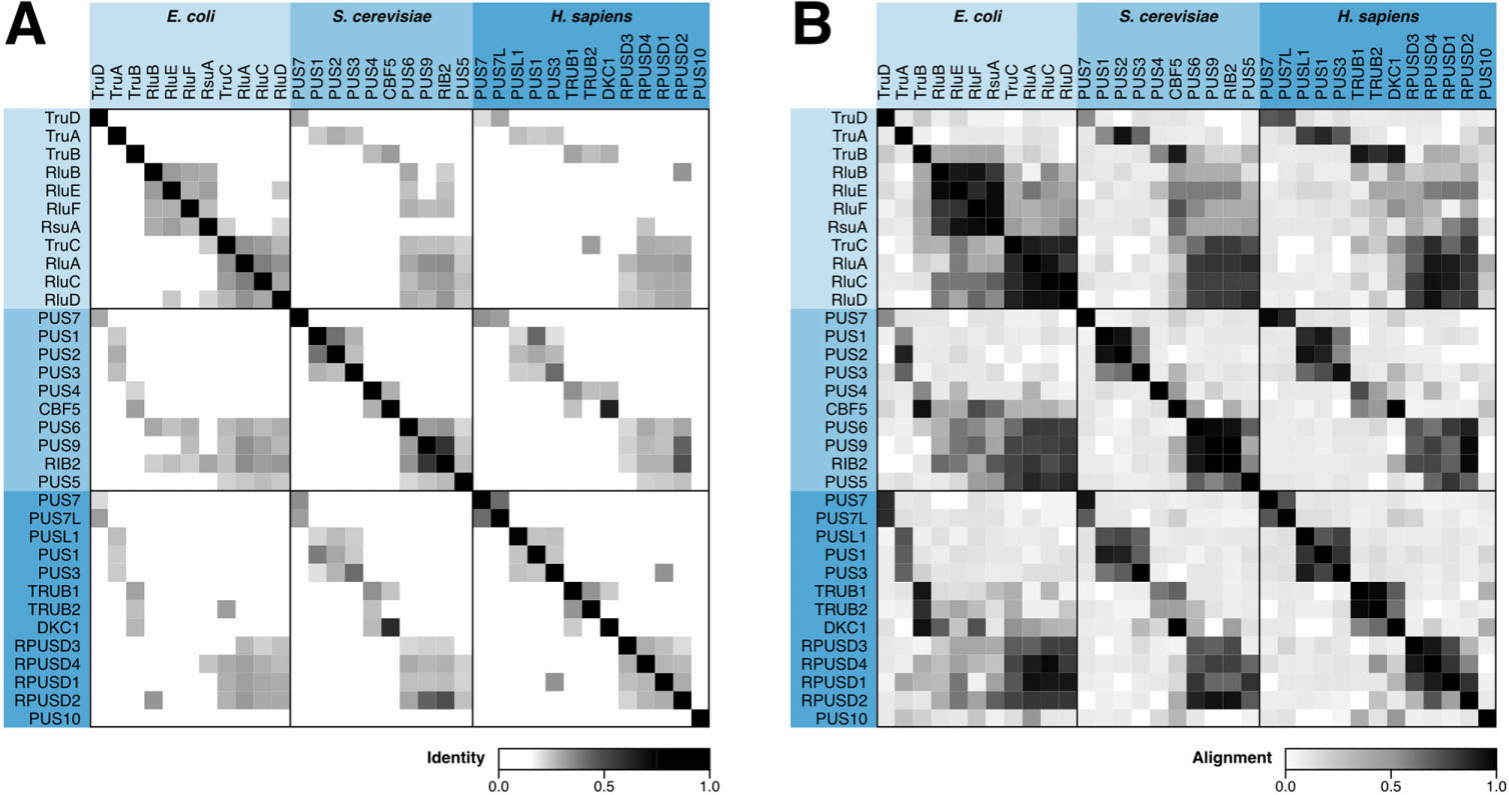
Amino acid sequence (**A**) and structural (**B**) alignment of PUS of *E. coli, S. cerevisiae*, and *H. sapiens*. The degree of primary sequence alignment is presented as identity percentage, and 3D alignment shown as proportion of pairwise matched and aligned residues. Sequence alignment was performed by BLAST.

Owing to the conservation of PUS and of their substrates across evolution, we hypothesized that a tentative matching of targets for the human enzymes could be achievable through *in silico* sequence and structural analysis. The amino acid sequences of all known and predicted PUS in *H. sapiens* were compared by alignment with those of the more extensively studied *S. cerevisiae* and *E. coli* (Fig. 1A and Supplementary Fig. S1A). Human counterparts of the RluA (RPUSD1, RPUSD2, RPUSD3, RPUSD4), TruA (PUS1, PUSL1, PUS3), TruB (DKC1, TRUB1, TRUB2), and TruD (PUS7, PUS7L) families are identified in this analysis; however, no candidates with remarkable sequence similarity to members of the RsuA family are identified, explaining in part (i) the lack of stand-alone enzymes for the pseudouridylation of the human cytosolic SSU 18S ribosomal RNA (rRNA), and (ii) the lack of detected Ψ residues in the human mtSSU 12S rRNA [52–57]. The pseudouridine synthase PUS10, present in archaea and eukaryotes but not in bacteria, clusters separately from the remaining enzymes, reflecting its divergent characteristics [58, 59].

While the number of members of the RluA family appears stable across *E. coli, S. cerevisiae*, and *H. sapiens*, other families have expanded: TruA increasing from one member in *E. coli* to three in *S. cerevisiae* and *H. sapiens*; TruD increasing from one member in *E. coli* and *S. cerevisiae* to two in *H. sapiens*; TruB increasing from one member in *E. coli* to two in *S. cerevisiae*, and three in *H. sapiens*.

Structural investigation was performed on the same set of enzymes, using X-ray diffraction crystal structures and predicted models generated by AlphaFold2 when the former is not available. As mentioned before, PUS have a core fold decorated by accessory domains, likely involved in setting substrate specificity. To avoid interference from these, only the core fold was considered for structural alignment (Fig. 1B). The results corroborate the findings obtained from sequence alignment in terms of family composition and support the absence of RsuA orthologues in yeast and human. Interestingly, and although PUS10 also clusters separately in this analysis, structural similarities to TRUB1 and DKC1 are observed. Additionally, proteins with topological similarity to bacterial, yeast and human PUS were searched across protein structure databases, further revealing a structural relationship between the bacterial TruA and human PUSL1 (Supplementary Fig. S1B).

In bacteria (*E. coli*), the RluA family targets tRNAs (RluA: U32; TruC: U65) and the LSU 23S rRNA (RluA: U746; RluC: U955, U2504, U2580; RluD: U1911, U1915, U1917) [60–65]. In turn, TruA is responsible for modifying U38, U39 and U40 in tRNAs, TruB pseudouridylates U55, TruC isomerises U65, and TruD does so on U13 [5, 63, 66–69]. In mitochondria, the mtLSU rRNA contains a single pseudouridine residue (16S Ψ1397 in human, equivalent to the bacterial 23S Ψ2580); this modification is performed by PUS5 in yeast, and RPUSD4 in mammals [8, 27, 28, 70]. On the other hand, multiple pseudouridine residues have been identified in mitochondrial tRNAs in universal positions 27a, 27, 28, 29, 31, 32, 39, 40, 50, 55, 57, and 67. Most of these modifications are present across several mt-tRNAs, while others have only been detected in single tRNAs (e.g. mt-tRNA^Asp^ Ψ31, mt-tRNA^Gln^ Ψ33, mt-tRNA^His^ Ψ35, mt-tRNA^Met^ Ψ50, mt-tRNA^Pro^ Ψ66 Ψ67, mt-tRNA^Ala^ Ψ68) [16, 20, 21, 66, 71–76].

It is known that the isoforms of PUS1 differentially localize in the cytoplasm and mitochondria, modifying U27 and U28 of tRNAs in the respective compartments, PUS3 modifies residues 38 and 39 of cytoplasmic tRNAs, and that members of the TruA family, harnessing the flexibility of their substrates, are able to modify uridine residues surrounding their main target. PUS7 modifies U13 in the D-arm stem of cytosolic tRNAs; no Ψ13 has been identified in human mt-tRNAs, despite this often being a U-rich region. TRUB1 and PUS10 modify U55 in cytoplasmic tRNAs, while TRUB2, which localizes to mitochondria, targets the same residue in the tRNAs of this compartment. The nucleolar protein DKC1 is the pseudouridine synthase in H/ACA snoRNPs for RNA-guided pseudouridylation of rRNA and telomerase RNA component (TERC), and thus is not expected to be relevant in the modification of mitochondrial transcripts. Although further evidence is needed, RPUSD1 and RPUSD2 do not seem to localize to mitochondria [77], and thus their relevance in the direct modification of mtRNAs may be limited. On the other hand, RPUSD3 and RPUSD4 have been shown to localize in mitochondria, with the former implicated in the modification of some mt-mRNAs, and the latter in the pseudouridylation of 16S mt-rRNA U1397 [26–28].

**Figure 2.**
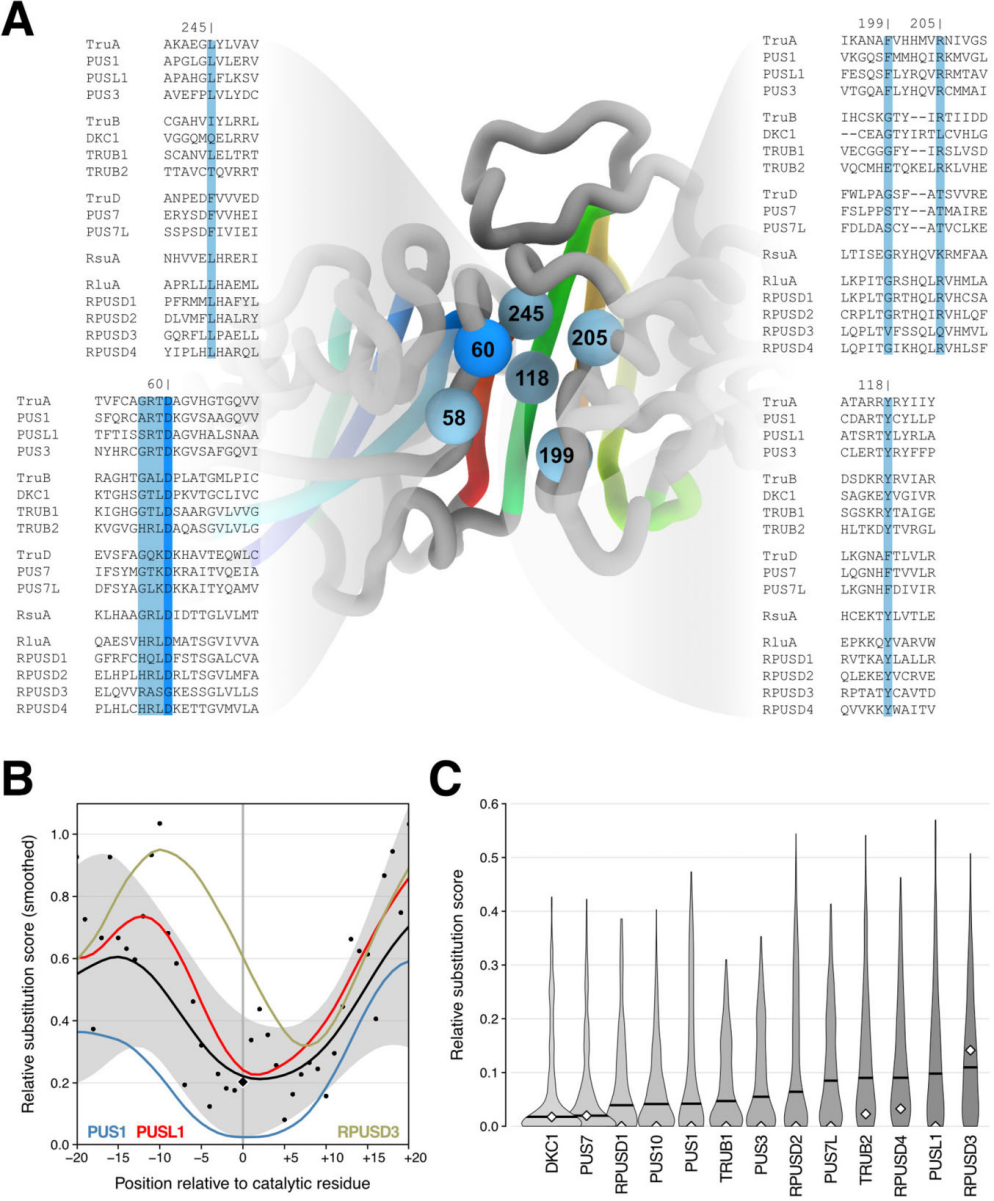
Conservation of functional residues of human PUS. (**A**) Alignment of relevant residues of human PUS. The head of each bacterial (*E. coli*) family is also included for phylogenetic reference. Selected conserved residues are highlighted in light blue, and the catalytic residue is highlighted in dark blue. Residues are contextualized in the structure of the *E. coli* TruA (PDB 1DJ0 [9]). Numbering is given relative to *E. coli* TruA. (**B**) Sequence variation of the catalytic site of human PUS. The smoothed average of the relative substitution score across the 13 human PUS based on sequence alignment is shown as a solid black line, with the associated standard deviation as a grey ribbon. The smoothed average of the relative substitution score is presented for PUS1 (solid blue line), PUSL1 (solid red line), and RPUSD3 (solid yellow line). Non-smoothed average relative substitution scores are presented for each assessed position as points. Residue positions are given relative to the catalytic aspartate residue, and its datapoint is presented as a diamond. (**C**) Distribution of substitution scores of all residues of human PUS. The median is presented for each protein as a black bar, and the scores for the residue annotated as or aligned to the catalytic residue are presented as white diamonds.

In addition to the catalytic aspartate residue, other residues in the active site of PUS are also highly conserved due to their role in catalysis [5]. Sequence and structural alignment of all investigated PUS in *E. coli, S. cerevisiae*, and *H. sapiens* identified the catalytic residue, which is an aspartate in all cases apart from human RPUSD3, where it is replaced by glycine, G135 (Fig. 2A and Supplementary Fig. S2A). The average relative substitution score [36] for the position corresponding to the catalytic residue is 1.8%, with RPUSD3 presenting the highest value, 14.2%, a value comparable to the average score for the whole protein, 13.3%, indicating a lower evolutionary substitution constraint (Fig. 2B and C). In addition, the position of the arginine responsible for flipping-out the substrate uridine (R62 in RluA, R151 in RPUSD4, equivalent to TruA R58) and the arginine forming a water bridge with the catalytic aspartate (R165 in RluA, R272 in RPUSD4, equivalent to TruA R205) are replaced by the biochemically distinct residues A133 and Q248, respectively (Fig. 2A and Supplementary Fig. S2B) [66, 78, 79]. Of note, the catalytic residue of PUS1 and PUSL1, D146 and D66, respectively, appear to be evolutionarily constrained to avoid substitutions. RPUSD3 has been identified within mitochondria and implicated in the pseudouridylation of some transcripts in this organelle [26, 27, 80]. Pending further investigation, and given the present evidence, it is unlikely that this enzyme directly performs pseudouridylation and may act in association with other, catalytically active, enzymes.

PUSL1 is a member of the eukaryotic TruA family, alongside PUS1 and PUS3. Given that PUS1 modifies cytosolic and mitochondrial tRNAs [4, 14–18], PUS3 does not show mitochondrial localization (not identified in MitoCarta 3.0 [80] and presents mainly cytosolic and nuclear localization [77]), and the presented enzyme—RNA modification correspondences, PUSL1 was considered as a candidate pseudouridine synthase responsible for the modification of U39 in mt-tRNAs.

### PUSL1 is a mitochondrial protein

**Figure 3.**
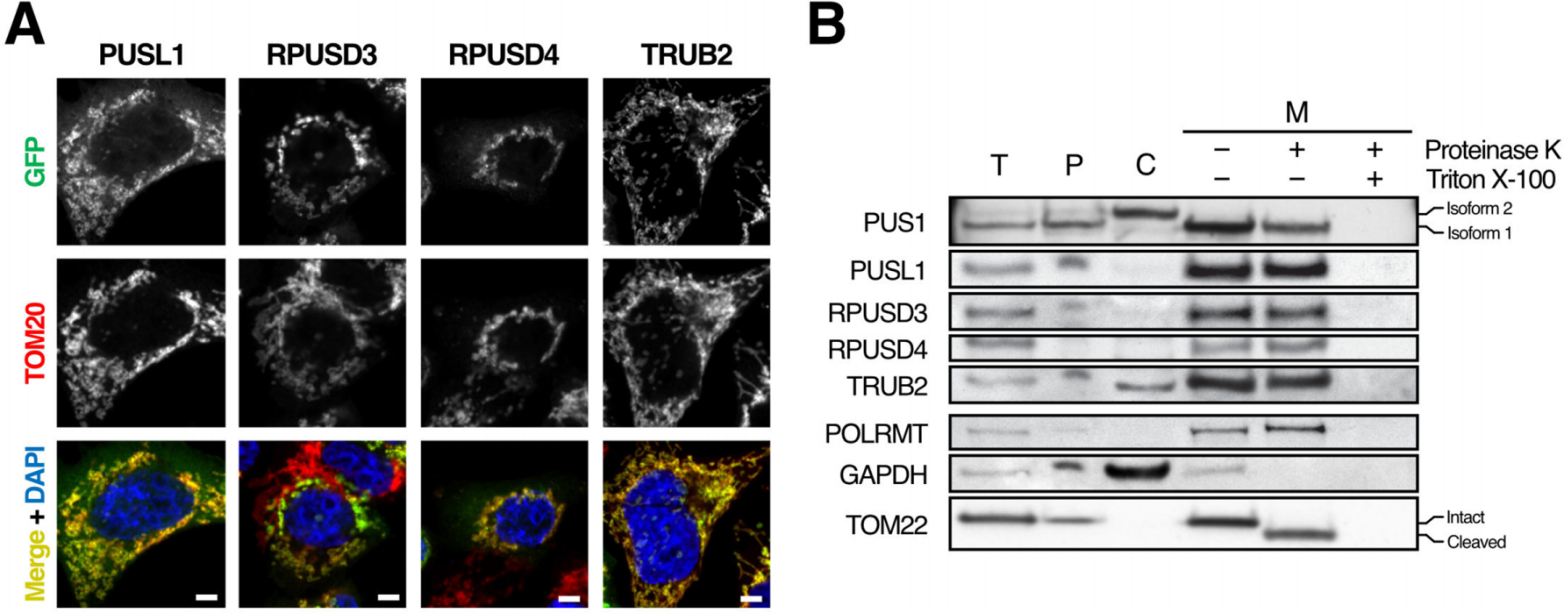
Subcellular localization of PUSL1 in human cells. (**A**) Confocal microscopy of cells expressing PUSL1 and other mitochondrial PUS with C-terminal GFP. TOM20 was used as a mitochondrial marker. Scale bar: 5 μm. (**B**) Biochemical fractionation of cell homogenates and immunodetection of PUSL1, other mitochondrial PUS and markers (POLRMT: mitochondrial matrix; GAPDH: cytosol; TOM22: outer mitochondrial membrane). T: total homogenate; P: homogenate pellet (non-lysed cells, cellular debris and nuclei); C: cytosol (post-mitochondrial fraction); M: mitochondrial fraction.

To determine the localization of PUSL1, we transiently expressed a C-terminus GFP-tagged fusion of this protein. The degree of co-localization of the GFP signal with that from the outer mitochondrial membrane transporter TOM20 indicates that PUSL1 is associated to mitochondria (Fig. 3A). To further investigate the presence of this protein within this organelle, purified mitochondria were incubated with proteinase K, which cleaves proteins that are not contained within the outer mitochondrial membrane and those exposed on its cytosolic side (e.g. TOM22). Being resistant to this treatment not only confirms that PUSL1 is associated with mitochondria, but also that, similarly to POLRMT and PUS1, it is present within this organelle (Fig. 3B).

### PUSL1 primarily modifies U39 of mt-tRNAs

Next, we sought to determine whether PUSL1 presents pseudouridine synthase activity and, if so, to identify its substrates and target residues. As mentioned before, PUS of the TruA family act on tRNAs; in mammalian systems, PUS1 is known to modify U27/U28 of both cytosolic and mitochondrial tRNAs, while PUS3 modifies U38/U39 of cytosolic tRNAs [81]. Given the homology of PUSL1 to TruA and the fact that residue 39 of mt-tRNAs is often pseudouridylated with no enzyme experimentally associated with this modification [82, 83], we interrogated the involvement of PUSL1 in the biogenesis of Ψ39 in mt-tRNAs.

To do this, mitochondrial-enriched RNA was treated with CMC, which chemically modifies guanine, inosine, uridine, 5-methyluridine and pseudouridine residues, followed by alkaline hydrolysis after which only Ψ-CMC adducts remain intact. CMC covalently binds to N3 of pseudouridine, altering the hydrogen-bonding pattern of the adduct; in addition, it is a bulky moiety (252.37 Da), thus constituting a steric hindrance to the processivity of reverse transcriptase [84, 85]. Harnessing these characteristics, pseudouridine residues were mapped at single-nucleotide resolution in mtRNA using a next-generation sequencing approach (mito-Ψ-Seq) [44]. This pipeline features the reverse transcription of CMC/HO^–^-modified RNA, whereby the enzyme tends to stall and/or fall-off from the RNA template during cDNA synthesis upon attempting to process a modified nucleotide leading to truncated cDNA fragments ending one nucleotide downstream to the modified sites. Thus, the 5′ of the generated fragments informing on the position of the modified residues and stoichiometry of the modification is calculated by generating PSI scores (i.e. calculating the RT-stop ratio that is the ratio between the number of reads originating from a truncated cDNA fragment and the number of overall reads covering the modified position)

**Figure 4.**
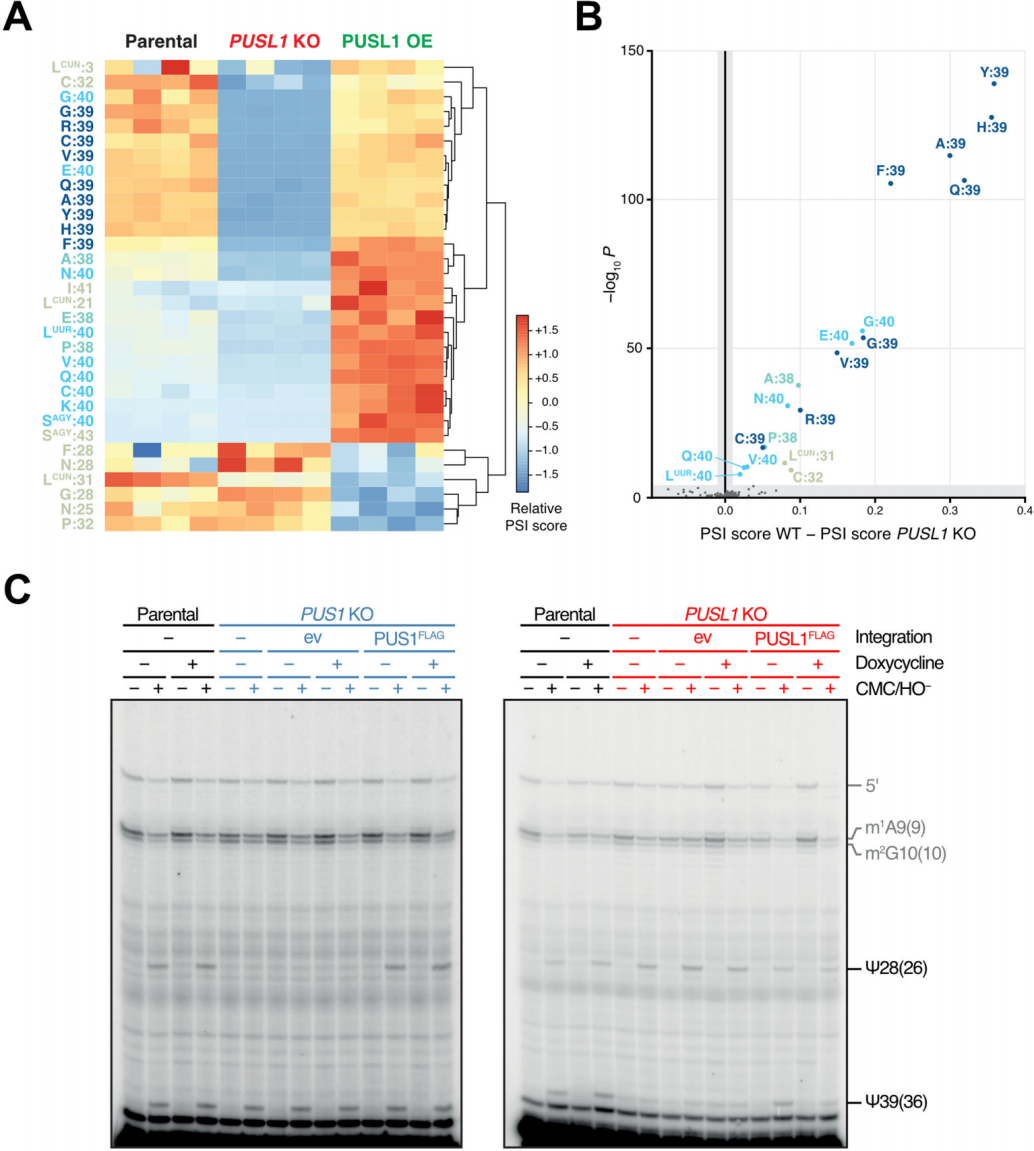
Mapping of PUSL1-dependent pseudouridine residues on mtRNAs. (**A**) mito- Ψ-Seq heatmap. Mt-tRNA residues for which pseudouridylation varies significantly upon ablation of PUSL1 and subsequent complementation are presented (four replicates). (**B**) Volcano plot of mito- Ψ-Seq results. Regions corresponding to results below statistical significance thresholds are shaded grey. (**C**) Analysis of mt-tRNA^Gly^ by CMC/HO^–^ RT-PEx. Autoradiograms of reverse transcription products separated in sequencing gels are presented. Annotations on the left are based on [72]. Related to Supplementary Fig. S4.

The identification of differentially pseudouridylated residues between knock-out (Supplementary Fig. S3) and parental epitranscriptomes provides an indication that PUSL1 is an active enzyme. The largest variation in pseudouridylation upon PUSL1 ablation is registered for universal position 39 of 9 mt-tRNAs: A, C, F, G, H, Q, R, V, Y (Fig. 4A and B). Inspecting all 22 mt-tRNAs, these 9 are all that contain a uridine residue in universal position 39. Furthermore, pseudouridylation of U39 in this subset of mt-tRNAs is nominally affected by complementation with overexpression of PUSL1. However, pseudouridylation of universal position 40 is detected in all mt-tRNAs containing a uridine residue in this position (C, E, G, K, L^UUR^, N, Q, S^AGY^, V) upon overexpression of PUSL1. Curiously, pseudouridylation of other residues shows a negative correlation with the increased titre of PUSL1, half of which are introduced by PUS1 (Ψ28 in mt-tRNA^Asn^, mt-tRNA^Phe^ and mt-tRNA^Gly^).

To corroborate these findings, we performed reverse transcriptase-mediated primer extension of radiolabelled primers specific to mt-tRNAs after CMC-treatment of RNA (CMC/HO^–^ RT-PEx). RNA from a PUS1 knock-out cell line was analysed in parallel with RNA from cells ablated of PUSL1 as an experimental control given that its targets are known [20]. As expected, pseudouridylation of universal positions 27 and 28 is abolished upon knock-out of *PUS1* (Fig. 4C and Supplementary Fig. S4). In line with results from mito-Ψ-Seq, Ψ39 was detected in mt-tRNA^Cys^, mt-tRNA^Phe^ and mt-tRNA^Gly^ (Fig. 4C and Supplementary Fig. S4). Furthermore, Ψ40 was detected in mt-tRNA^Cys^ upon complementation of the *PUSL1* knock-out cell line (Supplementary Fig. S4). The unique pseudouridylation of U50 in mt-tRNA^Met^ was tested and found not to be performed by PUSL1 as it is insensitive to the depletion of this protein. Curiously, signal for a CMC-sensitive, PUS1- and PUSL1-independent modification is observed for this mt-tRNA in a position close to 27, typically attributable to PUS1; mass spectrometric analysis identifies Ψ27, f^5^C34, Ψ50 and Ψ55 as the only modifications of the human mt-tRNA^Met^ [72], which further narrows down the possibility of this signal being originated by Ψ27.

Altogether, this shows that (i) PUSL1 is an active enzyme, (ii) the primary target of PUSL1 is universal residue 39, (iii) all mt-tRNAs with U39 are substrate of PUSL1, likely with structural determinants over sequence specificity, (iv) PUSL1 can modify neighbouring positions 38 and 40 in a dose-dependent manner, and suggests that (v) pseudouridylation of additional positions, performed by other enzymes, may be regulated by the titre of PUSL1.

### Ablation of *PUSL1* does not impair homeostatic mitochondrial translation

Given that PUS1 and PUSL1 are involved in RNA modification within mitochondria, the effect of their depletion on mitochondrial function was assessed. Firstly, cell proliferation was assessed when cells were cultured in media containing high or low concentrations of glucose, as well as when glucose was replaced by galactose [86]. While the median maximum proliferation rate of parental cells is 2.7% h^−1^ in permissive conditions (high glucose), *PUS1* and *PUSL1* knock-out cell populations expand significantly slower (2.4% and 2.2% h^−1^, respectively) (Supplementary Fig. S5A). The same trend is observed for parental (1.0% h^−1^) and *PUSL1* knock-out (0.6% h^−1^) populations in galactose medium, indicating that the latter are unable to supply enough energy from mitochondria-related metabolic pathways to sustain cellular activities at homeostatic rates. As expected, an extreme case of this scenario is observed in the same medium when mitochondrial dysfunction is induced by chloramphenicol inhibition of organellar translation. This compound also produces an effect on cell proliferation in glucose media, leading to a more pronounced retardation when glucose concentration is lower (~43%, 49%, and 37% reduction in maximum proliferation rate in parental, *PUS1*, and *PUSL1* knock-out cells, respectively, when comparing non-supplemented and 200 µg ml^−1^ chloramphenicol low glucose media, versus 25%, 21%, and 26% in high glucose media), as a result of the more stringent reliance on mitochondrial function.

Residue U39 is pseudouridylated in 9 of the 22 mt-tRNAs [72], and is involved in anti-codon stem base pairing, which may indicate a potential role of this RNA modification in the structural equilibrium and participation of mt-tRNAs in translation. To test this hypothesis, we investigated whether lack Ψ39 can disrupt mitochondrial translation. For this, nascent mitochondrial peptides were metabolically labelled by incorporation of [^35^S]-methionine in the presence of a cytosolic translation inhibitor. Autoradiography shows comparable profiles of *de novo* synthesized proteins in both *PUS1* and *PUSL1* knock-out cells and the parental cell. This suggests that PUS1 and PUSL1, as well as the modifications they introduce in mtRNAs, have no direct or obvious implication in mitochondrial translation in the investigated cell type and applied conditions (Supplementary Fig. S5B).

### *PUSL1* is associated with human pathology

**Figure 5.**
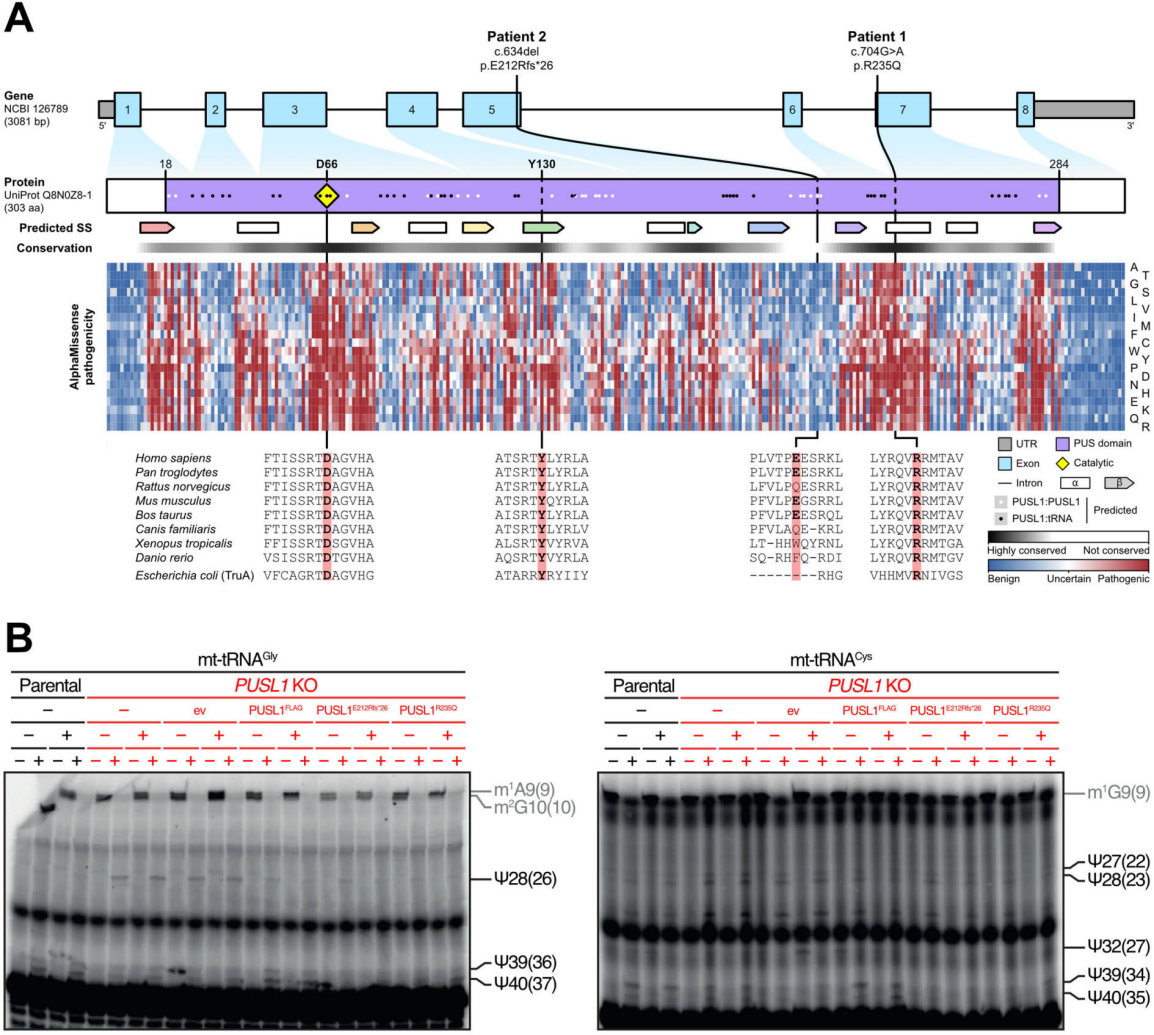
Molecular effects of PUSL1 p.Glu212Argfs*26 and p.Arg235Gln variants identified in patients. (**A**) Gene structure of *PUSL1* and localization of amino acid residues affected by mutations. PUS domain was annotated with information from InterPro. Secondary structure and dimerization interfaces were predicted using AlphaFold2, and PUSL1:tRNA interactions were guided by inspection of PDB 2NR0. Conservation scores were obtained from Aminode. (**B**) Assessment of pseudouridylation of U39 in mt-tRNAs by CMC/HO^–^ RT-PEx. Autoradiograms of reverse transcription products separated in sequencing gels are presented. Annotations on the left are based on [72].

While PUS1 has been linked with human pathology, particularly the mitochondrial disorder MLASA (Myopathy, Lactic Acidosis, Sideroblastic Anaemia) [19–21, 25, 87–89], the role of other PUS in human disease remains largely unexplored. We studied two patients who developed mitochondrial disease symptoms: Patient 1, born to consanguineous parents, showed signs of weakness, cyanosis, and hypotonia at birth following an *E. coli* infection and later experienced delays in development, leading to significant complications such as cerebellar ataxia and microcephaly by age 11 (further details in Supplementary Data S2). Whole Exome Sequencing (WES) analysis identified a homozygous variant NC_000001.11:c.704G > A (p.Arg235Gln) in the *PUSL1* gene. According to the gnomAD database, the allele frequency for *PUSL1* c.704G > A is 8.54 × 10^−5^, with no homozygotes identified. This substitution has a CADD score of 32, PolyPhen-2 score of 0.993, classified as ‘probably damaging’, and SIFT score of 0 that is predicted to ‘affect protein function’, indicating perturbations in the structure and function of the mutated proteins. Patient 2, a 68-year-old man, developed early dementia due to severe vascular leukoencephalopathy from age 60. Imaging showed arterial stenosis and microbleeds, with ineffective immunosuppressor treatment. He had microcephaly (<1st percentile) and lifelong intellectual disability, unable to manage finances or read independently. WES analysis revealed a homozygous NC_000001.11:c.634del, p.Glu212Argfs*26 variant (Fig. 5A) (further details in Supplementary Data S2).

Some of the previously reported cases of MLASA harbour the *PUS1* c.883C > T [24] or c.884G > A [23] variants, which translate into the substitutions R295W or R295Q, respectively. The affected residue in these cases is structurally equivalent to R235 in PUSL1, which is altered to a glutamine in Patient 1. Residue R235 of PUSL1 has been conserved throughout evolution (Aminode substitution score of 0) across most pseudouridine synthase families (Fig. 2) and is positioned in the catalytic cleft of the enzyme, opposite of the catalytic residue D66 (Supplementary Fig. S2B). It is structurally equivalent to TruA R205 and may contribute to catalysis by establishing electrostatic interactions with the catalytic residue, although not determinant for substrate binding [90].

While the effect of Arg235Gln is relatively easy to rationalize in the context of the structure and function of PUSL1, the same is not true for the p.Glu212Argfs*26 variant as it not only involves mutation of multiple residues but also truncation of the encoded protein. To better analyse this, the structure of the PUSL1 Glu212Argfs*26 variant was predicted using AlphaFold. Remarkably, part of the 26 amino acids encoded downstream of the frameshift are able to form a β-strand, positioned in the same manner as in the wild-type PUSL1 (residues 218–226 in PUSL1 and neo-residues 221–229 in PUSL1 Glu212Argfs*26); this is likely permitted by some redundancy in the altered sequence stretch where some of these alterations are structurally silent: W220, L222 and S226 in wild-type PUSL1, and neo-residues W222, L224, and S229 in PUSL1 Glu212Argfs*26. The truncation caused by the frameshift renders the variant devoid of two helices, decorations to the non-RRM half of the core fold and potentially involved in substrate binding/recognition, and a C-terminal strand, which forms the back of the catalytic cleft and is the key point of connection between the two halves of the core fold. Without this, the two halves of PUSL1 Glu212Argfs*26 may fail to attain a stable structure that allows the interaction with the substrate RNAs, providing a possible explanation for the lack of pseudouridylation in U39 of mt-tRNAs.

To study these potentially pathogenic variants of PUSL1, cells knocked-out for this gene were complemented with the cDNA encoding the same variations. As a functional read-out, pseudouridylation of mt-tRNAs was assessed by CMC/HO^–^ RT-PEx. In line with these predictions, it was observed that complementation with PUSL1 p.Glu212Argfs*26 does not restore pseudouridylation of U39 (Fig. 5B). On the other hand, PUSL1 p.Arg235Gln presented some residual function, potentially due to impaired but not completely abolished catalytic activity. Taken together, PUSL1 is implicated in human pathology, as evidenced by identified pathogenic variants in two patients with mitochondrial disease symptoms. Although the available genetic and functional evidence supports a role for PUSL1 dysfunction in the disease presentations described here, definitive assignment of causality remains challenging given the limited availability of patient-derived material and the subtle cellular phenotype.

## Discussion

Recent works have demonstrated the presence of a cohort of enzymes with pseudouridine synthase within mitochondria. The most studied member, PUS1, has been linked to human pathology, highlighting the relevance of this topic, which remains to be fully characterized and understood. Here, we investigated the mitochondrial epitranscriptome, focusing on pseudouridylation and the associated molecular players. Taking into account the biological constraints of the mitochondrial system, as well as experimental report of proteins with pseudouridine synthase activity in this organelle, we assessed the existence of additional enzymes in this cohort, and hypothesized PUSL1 to be a plausible candidate. Using a combination of bioinformatics tools, and drawing on the conservation of PUS (Figs 1 and 2, and Supplementary Fig. S1), we were able to predict that PUSL1, as a human orthologue of truA, modifies uridine residues in universal position 39 of tRNAs, with potential to also modify neighbouring uridine residues.

We show that PUSL1 preferentially localizes in mitochondria (Fig. 3), it has pseudouridine synthase activity *in vivo* and, as predicted, modifies universal residue 39 in all mt-tRNAs containing a uridine in this position (mt-tRNAs A, C, F, G, H, N, R, V, Y) (Fig. 4 and Supplementary Fig. S4). Furthermore, we show that, despite the lack of pseudouridylation, steady-state mitochondrial translation is not significantly affected under homeostatic conditions upon ablation of PUSL1 or PUS1 (Supplementary Fig. S5). The absence of a pronounced phenotype in HEK293 cells does not exclude physiological relevance, as the functional consequences of RNA modification defects frequently emerge only in differentiated tissues, under stress conditions, or in sensitized genetic backgrounds.

Individual ablation of PUS1 or PUSL1 does not lead to an alteration in the level of pseudouridylation of U39 or U27/U28, respectively. This indicates that, despite both being truA orthologues, the function of these human enzymes is not redundant, and they target specific and non-overlapping substrates in mt-tRNAs. Additionally, this lack of redundancy in homeostatic conditions may imply a non-essentiality for these RNA modifications in the absence of stress factors. The relatively subtle phenotype observed upon loss of PUSL1 is consistent with previous studies of tRNA modification enzymes [91–97], where disruption of individual modifications often produces minimal effects under standard growth conditions and becomes functionally relevant only in specific physiological or stress contexts.

Mapping of pseudouridine residues raised some unexpected results. First, overexpression of PUSL1 was seen to result in ectopic modification of the target-adjacent U40 (mt-tRNAs C, E, G, K, L^UUR^, N, Q, S^AGY^, V) and U38 (mt-tRNAs A, E, P) (Fig. 4A–C and Supplementary Fig. S4). This may be a consequence of substrate flexibility, as similar cases have been reported for truA, where the flexibility of the anticodon stem-loop correlates with modification efficiency [9, 66]. Interestingly, an uridine residue in universal position 39 is not required for U40 to be modified, as evidenced by the case of mt-tRNA^Leu(UUR)^ and mt-tRNA^Ser(AGY)^, indicating that the nucleoside at residue 39 is not a strong determinant for PUSL1 binding and catalysis.

While box H/ACA snoRNPs contain an RNA component that guides the pseudouridine synthase activity of the complex, standalone enzymes rely on their conformation, inherent flexibility and presence of additional domains to recognize their target, likely in a structure- rather than in a sequence-dependent manner. PUS1 is an example of this, lacking a strict substrate sequence consensus but presenting a strong structural motif, which is sufficient for substrate modification at the 5′ end of stem loops [18, 98]. During the revision of this paper, Xu *et al*. published evidence for modification of U39-40 in mt-tRNAs by PUSL1, with no clear sequence motif [99].

Despite being a eukaryotic orthologue of the bacterial enzyme truA, PUS1 has a C-terminal helical extension located at the back and top of the core β-sheet (Supplementary Fig. S6A and B), which alters the tRNA binding configuration and prevents PUS1 homodimerization, thus accounting for their different substrate preferences [9, 78, 100, 101]. However, PUSL1 does not contain such an extension, and is structurally more similar to truA in that aspect, explaining the targeting of similar substrates [66]. In addition, residues 38–40 of bound tRNAs are placed near the active site clefts of the truA homodimer, explaining the potential for their modification in case these are uridines [9, 66].

Another unexpected result was observed in the CMC/HO^–^ RT-PEx mapping performed for mt-tRNA^Met^ (Supplementary Fig. S4). While Ψ55 did not show a dependency on the presence of PUS1 or PUSL1, a hydrolysis-insensitive adduct band was present across all CMC/HO^–^-treated samples; this band should correspond to universal position 27 and, based on this, the enzyme responsible for its modification is expected to be PUS1. However, this band was also observed in *PUS1* knock-out samples.

It has been shown that the mitochondrial PUS RPUSD3, RPUSD4 and TRUB2 not only are essential for OxPhos function, but are also encoded by essential genes. These three proteins are hypothesized to be associated with FASTKD2, NGRN, and WBSCR16 in a ‘functional module’ that regulates mitochondrial translation, possibly at the level of mitoribosome biogenesis [26]. The bacterial and yeast proteins presenting the highest sequence identity to human RPUSD3 are rluC and PUS9, respectively, which are involved in the modification of U32 in tRNAs. Additionally, PUS9 localizes in the mitochondria of yeast [102]. Our results (Fig. 2 and Supplementary Fig. S2) point towards an evolutionary inactivation of RPUSD3 given that the catalytic site motif RLD has been altered, with the catalytic aspartate being replaced by glycine. Given that some human mt-tRNAs present Ψ32 (mt-tRNA^Cys^, mt-tRNA^Pro^, mt-tRNA^Arg^, whereas U32 of mt-tRNA^Ala^, mt-tRNA^Glu^, mt-tRNA^His^ and mt-tRNA^Gln^ do not appear to be pseudouridylated [72]), it is possible to hypothesize that (i) RPUSD3 can still be active by using an alternative set of residues for catalysis, (ii) the overall spatial arrangement of RPUSD3 is different from the predicted, or (iii) RPUSD3 is inactive and it has been functionally replaced by other enzyme(s), potentially of the RluA family. Independent experimental evidence confirms the lack of *in vivo* pseudouridine synthase activity of RPUSD3 [99].

The large subunit of mitochondrial ribosomes contains a structural tRNA in place of the 5S rRNA. In humans, this is typically mt-tRNA^Val^; however, depending on the availability and stability of this tRNA, mtLSU can incorporate mt-tRNA^Phe^ instead [103]. Our results show that both mt-tRNA^Val^ and mt-tRNA^Phe^ are substrates of PUSL1, orthogonally corroborating RNA mass spectrometry findings [72]. Additionally, mt-tRNA^Phe^ is also a substrate of PUS1, which modifies U28 [29]. In the context of the mtLSU, the anticodon stem of the structural tRNA is positioned in a constriction formed by uL18m and mL38, which contact residue 39, and more distally by mL40 and mL48, which contact residue 28. Of note, the mL40:mL46:mL48 module in the head region of the mtLSU shares an extensive interface with the tRNA, which bridges it to uL18:bL27:mL38 and to the body of the mtLSU. The protein-protein contacts between these two sets of mitoribosomal proteins is limited, and it can thus be hypothesized that structural alterations of the anticodon stem of mt-tRNA^Val^ and mt-tRNA^Phe^ can have implications in the conformation of the mtLSU head, with a potential impact in translation.

PUS1 mutations have been associated with MLASA [19–21, 25, 87–89]. Although no major mitochondrial translation defects were observed in this study upon depletion of PUS1 or PUSL1 (Supplementary Fig. S5), determining whether the same still holds in more physiological and non-homeostatic conditions could help elucidating the role if this and other RNA modifying enzymes. Furthermore, mutations identified in human patients revealed potential impact on the activity of PUSL1 (Fig. 5), and consequently contributing to the clinical presentation. Taking into consideration that highly deleterious mutations are also highly unlikely to be detected, the gnomAD database was searched for further potentially pathogenic missense mutations in PUS. The conserved tyrosine residue at the bottom of the catalytic cleft (structurally equivalent to *E. coli* TruA Y118) is rarely found substituted to cysteine with a frequency of 2.4 × 10^−5^ in PUS1 (Y201) and 1.6 × 10^−6^ for PUSL1 (Y130). Substitution of the arginine residues in the catalytic cleft is also rare (combined frequencies of 3.2 × 10^−5^ for PUS1 R144, and 1.2 × 10^−5^ for PUSL1 R64, structurally equivalent to *E. coli* TruA R58). This is in line with combined frequencies of 1.2 × 10^−5^ for PUS1 R295, the residues where pathogenic changes were detected previously [24], and 1.5 × 10^−4^ for PUSL1 R235, reported in this work. All of these substitutions present a CADD score above 20, PolyPhen-2 score of 0.993–1 that classifies as ‘probably damaging’, and SIFT score of 0 that is predicted to ‘affect protein function’, indicating perturbations in the structure and function of the mutated proteins; PUS1 R144W is further classified as ‘pathogenic/likely pathogenic’ by ClinVar. As expected, mutation of the catalytic aspartate is rare (frequencies of 1.6 × 10^−6^ and 1.5 × 10^−6^ for PUS1 D146E and PUSL1 D66Y, respectively) and share the same aforementioned pathogenicity prediction scores. The rarity of these missense *PUSL1* variants, especially those located in critical residues of the catalytic cleft, coupled with their high pathogenicity prediction scores, underscores the potential for these variants to be clinically meaningful by significantly impairing enzymatic function.

We acknowledge that the two patients described in this study present with clinically distinct neurological phenotypes and that neither fulfilled classical diagnostic criteria for a definitive mitochondrial disorder. This clinical heterogeneity, together with the relatively subtle cellular phenotype observed upon PUSL1 ablation in cultured cells, complicates direct genotype–phenotype interpretation. However, marked phenotypic variability is a well-recognized feature of mitochondrial disease and has also been reported for defects affecting mtRNA modification pathways, including TRMT5-associated disorders, where presentations range from cardiomyopathy and developmental delay to neuropathy, spastic paraparesis, portal hypertension, and exercise intolerance. In this context, the identification of rare homozygous PUSL1 variants in two unrelated patients with neurological disease, combined with the demonstrated impairment of mt-tRNA pseudouridylation caused by these variants, supports the view that defective PUSL1 function contributes to human pathology. At the same time, we cannot exclude the possibility that additional genetic, environmental, or tissue-specific modifiers influence the clinical presentation and severity associated with PUSL1 dysfunction [104–108].

Knowledge on the role of mitochondrial pseudouridylation has been developing but is not yet complete. For that, it will be crucial to identify the enzymes responsible for the remaining modified sites in tRNAs, determine the landscape of pseudouridines in mRNAs as well as its dynamicity under different cellular conditions, and ultimately understand the role of these proteins in the context of mitochondrial homeostasis and dysfunction.

## Conflict of interest

M.M. is a founder, shareholder, and member of the Scientific Advisory Board of Pretzel Therapeutics, Inc. The remaining authors declare no competing interests.

## Supplementary Material

gkag805_Supplemental_Files

## Data Availability

Mito-Ψ-Seq data have been deposited in the Gene Expression Omnibus (GEO) database with accession number GSE281373. Codes for the analyses described in this paper are available at https://github.com/SchwartzLab/mazter_mine. Minor updates include: https://doi.org/10.5281/ZENODO.3581426 (2019)—for the bam3ReadEnds.R code, https://github.com/aldemasas/pseudouridine—for calculation of RT-stop ratios, and https://github.com/aldemasas/ac4c-seq—for statistical testing. This is also available on Zenodo at https://zenodo.org/records/21533694 for aldemasas/ac4c-seq, https://zenodo.org/records/21533728 for aldemasas/pseudouridine, and http://doi.org/10.5281/zenodo.3581426 for SchwartzLab/mazter_mine.
