# Supplementary material for: The pseudouridine synthase PUSL1 modifies U39 of mitochondrial tRNAs and is linked to human neurological phenotypes
Source: Nucleic Acids Res. 2026 Aug 14;54(15):gkag805. doi: 10.1093/nar/gkag805 (PMC13473875; doi:10.1093/nar/gkag805)
Supplement: Supplementary file 1 [file gkag805_supplemental_files.zip › Rebelo-Guiomar2024NAR Supplementary Information MAM240626 .docx]

**Supplementary Information**

**The pseudouridine synthase PUSL1 modifies U39 of mitochondrial tRNAs and is linked to human neurological phenotypes**

Pedro Rebelo-Guiomar, Aldema Sas-Chen, Nadav Kra-Oz, Christopher Powell, Guillaume Jouret, Morgane Plutino, Veronique Paquis-Flucklinger, Schraga Schwartz and Michal Minczuk

**Supplementary figure legends**

Supplementary Figure S1. Alignment of amino acid sequences (A) and structural search (B) of pseudouridine synthases of *Escherichia coli*, *Saccharomyces cerevisiae* and *Homo sapiens*. The degree of primary sequence alignment is presented as identity percentage, and the degree of structural matching is presented as TM-score. Sequence alignment and structural search were performed by Clustal Omega (108) and FoldSeek (41), respectively.

Supplementary Figure S2. Conservation of residues of the catalytic site of pseudouridine synthases, with focus on RPUSD3. (A) Sequence alignment of RPUSD3 and RPUSD4 in reference species. Sequence of members of the bacterial RluA family, to which RPUSD3 is orthologue, are also aligned for reference. The position of the catalytic aspartate is highlighted in dark blue in the sequence alignment. (B) Structural alignment of the catalytic aspartate (blue highlight) and auxiliary arginine residues. The crystal structures of *E. coli* TruA (PDB 1DJ0 (9)), RluA (PDB 2I82 (78)), and *H. sapiens* PUS1 (PDB 4J37 (77)) and RPUSD4 (PDB 5UBA (109)), as well as structural predictions of *H.* sapiens PUSL1 and RPUSD3 (38) are presented, focusing on their catalytic cleft (catalytic aspartate and intercalating arginine residues and their structural equivalents are labelled). RluA was co-crystallised with a pseudo-substrate containing a 5-fluorouridine residue, flipped-out into the active site, which is also shown.

Supplementary Figure S3. Validation of knock-out and complemented cell lines. Immunodetection of PUS1 and PUSL1 was performed on CRISPR/Cas9-generated knock-out and parental (Par) cell lines. Knock-out lines complemented with empty vector (ev) or with cDNA for the ablated gene (encoding a C-terminal FLAG tag) were inspected for doxycycline-induced expression of the transgenes. Short and long exposures are presented.

Supplementary Figure S4. Mapping of pseudouridine residues on mt-tRNA^Phe^, mt-tRNA^Cys^, and mt-tRNA^Met^ by CMC/HO^–^ RT-PEx (continuation of Figure 4C). Autoradiograms of reverse transcription products separated in sequencing gels are presented. Annotations on the left are based on (71).

Supplementary Figure S5. Cellular effects of *PUSL1* ablation. (A) Maximum proliferation rate of parental, *PUS1* and *PUSL1* knock-out cells in medium containing glucose (permissive) or galactose (restrictive). (B) Metabolic labelling of *de novo* translated mitochondrial proteins (top left: autoradiogram; top right: autoradiogram profile). Coomassie brilliant blue (CBB) staining is shown as a loading indicator. This experiment was replicated three times with similar results, as shown.

Supplementary Figure S6. (A) Analysis of the dimeric structure of TruA, PUS1 and PUSL1. Assessment of predicted (AlphaFold 3) dimeric structures by pTM (predicted template modelling, overall fold) and ipTM (interface predicted template modelling, inter-subunit relative position) scores. Suggested thresholds are shown as dark grey lines. Predicted alignment error (PAE) plots are presented, scaled to protein length, for the best model of each entry (darker green represents lower PAE). (B) Predicted dimeric structures. The best predicted structure of each entry is presented (unstructured N- and C-termini were removed for the sake of simplicity of representation). The C-terminal region of each protomer is highlighted in orange.
