# Supplementary material for: The pseudouridine synthase PUSL1 modifies U39 of mitochondrial tRNAs and is linked to human neurological phenotypes
Source: Nucleic Acids Res. 2026 Aug 14;54(15):gkag805. doi: 10.1093/nar/gkag805 (PMC13473875; doi:10.1093/nar/gkag805)
Supplement: Supplementary file 1 [file gkag805_supplemental_files.zip › SupplementaryData2_MAM121124.docx]

**Supplementary Data 2**

**Clinical characterisation of the patients with *PUS1L* variants**

**Patient 1** is the first son of consanguineous parents. His younger brother is healthy. He was born at 36 weeks of gestation with a birth weight of 2.4 kg (25^th^ percentile), a height of 45 cm (10^th^ percentile), and a head circumference of 32 cm (25^th^ percentile) in the context of an *Escherichia coli* materno-fetal infection. At birth, he exhibited neonatal weakness, cyanosis, and hypotonia. A transfontanellar ultrasound and an electroencephalogram conducted at that time were normal. The overall disease progression was marked by psychomotor delay – he achieved the ability to sit at 1 year and to walk at 2.5 years. Over time, he developed progressive encephalopathy characterized by symptoms including cerebellar ataxia (ataxia and dysmetria) at 18 months, loss of ambulation by 6 years, and cerebellar atrophy, as seen on a brain MRI at age 7. A clinical examination at Necker Hospital at age 9 identified microcephaly, cerebellar ataxia, autistic features with hyperactivity, and inappropriate laughter. Further biochemical tests revealed an elevated lactate/pyruvate (L/P) ratio of 26. A muscle biopsy indicated normal histology; however, mitochondrial respiratory chain complex spectrophotometry was non-contributory. By age 11, the patient experienced episodes of weakness accompanied by dyspnoea, cyanosis, pallor, and excessive sweating. Neuroimaging showed severe atrophy of the posterior fossa, brainstem, and the pons on brain MRI. Despite these symptoms, spectrophotometric, polarographic, and Blue Native (BN) PAGE analyses on skin biopsy material were normal. Whole Exome Sequencing (WES) identified a homozygous c.704G>A, p.R235Q variant in the *PUSL1* gene. The segregation was in favour of the pathogenicity of this variant, found in the heterozygous state in the parents and the healthy brother. The parents declined a second muscle biopsy, and no additional usable tissue samples are available for further analysis.

**Patient 2** is a 68-year-old man, initially referred to the department of clinical genetics for etiologic assessment of early dementia, in a context of severe vascular leukoencephalopathy evolving since the age of 60 years. Brain imaging revealed distal arterial stenosis with occasional development of collateral circulation, severe vascular leucoencephalopathy, micro- and macrovascular ischemic sequels and micro bleeds. There was no argument strongly suggestive of cerebral vasculitis, and an immunosuppressor treatment was ineffective. During the clinical genetics’ consultation, in addition to this vascular condition, the patient was found to have a head circumference measured at 53 cm, corresponding to microcephaly (<1^st^ percentile). Moreover, the relatives reported that before his dementia, the patient had never been able to independently manage his finances, could not read, and had language difficulties, suggesting an intellectual disability preceding the cerebrovascular pathology. However, he was able to work in a conventional environment, and his work consisting of repeating simple tasks. The patient has a sister with a compatible symptomatology, combining an initial limited autonomy suggestive of neurodevelopmental delay and then secondarily the occurrence of an early and unusually pronounced vascular leucoencephalopathy. WES analysis revealed a homozygous c.634del, p.E212Rfs*26 variant. Skin or muscle biopsy was not available for this patient.
